# Modeling Discontinuous Cultural Evolution: The Impact of Cross-Domain Transfer

**DOI:** 10.3389/fpsyg.2022.786072

**Published:** 2022-02-24

**Authors:** Kirthana Ganesh, Liane Gabora

**Affiliations:** Department of Psychology, University of British Columbia, Kelowna, BC, Canada

**Keywords:** autocatalytic network, cross-domain transfer, cultural evolution, cultural discontinuity, Hohlenstein-Stadel Löwenmensch figurine, inspiration, music, sculpture

## Abstract

This paper uses autocatalytic networks to model discontinuous cultural transitions involving cross-domain transfer, using as an illustrative example, artworks inspired by the oldest-known uncontested example of figurative art: the carving of the Hohlenstein-Stadel Löwenmensch, or lion-human. Autocatalytic networks provide a general modeling setting in which nodes are not just passive transmitters of activation; they actively galvanize, or “catalyze” the synthesis of novel (“foodset-derived”) nodes from existing ones (the “foodset.”) This makes them uniquely suited to model how new structure grows out of earlier structure, i.e., cumulative, generative network growth. They have been used to model the origin and early evolution of biological life, and the emergence of cognitive structures capable of undergoing cultural evolution. We conducted a study in which six individual creators and one group generated music, prose, poetry, and visual art inspired by the Hohlenstein-Stadel Löwenmensch, and answered questions about the process. The data revealed four through-lines by which they expressed the Löwenmensch in an alternative art form: (1) lion-human hybrid, (2) subtracting from the whole to reveal the form within, (3) deterioration, and (4) waiting to be found with a story to tell. Autocatalytic networks were used to model how these four spontaneously derived through-lines form a cultural lineage from Löwenmensch to artist to audience. We used the resulting data from three creators to model the cross-domain transfer from inspirational source (sculpted figurine) to creative product (music, poetry, prose, visual art). These four spontaneously-generated threads of cultural continuity formed the backbone of this Löwenmensch-inspired cultural lineage, enabling culture to evolve even in the face of discontinuity at the level conventional categories or domains. We know of no other theory of cultural evolution that accommodates cross-domain transfer or other forms of discontinuity. The approach paves the way for a broad scientific framework for the origins of evolutionary processes.

## 1. Introduction

This paper provides an existence proof^1^ that a formal model of cultural evolution can accommodate a widespread form of cultural discontinuity: cross-domain transfer. In *cross-domain transfer*, an inspirational source from one domain (e.g., music) influences a creative work in another (e.g., painting) (Gabora, 2010b; Gabora et al., 2012; Ranjan et al., 2013; Scotney et al., 2019). For example, George Mestral's invention of Velcro was inspired by analogy to burdock root seeds (Freeman and Golden, 1997) which, in turn, inspired “shoelace-less runners” (or “shoelace-less sneakers.”) This example illustrates a central feature of cross-domain transfer: with respect to the most obvious techniques for classifying them—e.g., as sculptures, pieces of music, or technological inventions—there is a discontinuity from one cultural output to the next. This research set out to test the hypothesis that there are nevertheless identifiable threads of continuity that connect the inspirational source with any cultural outputs it inspires, which serve as the basis for their proximity in a cultural lineage, enabling us to formally model even seemingly discontinuous lines of cultural descent.

The term *culture* generally refers to extrasomatic adaptations, including behavior and artifacts, that are socially rather than genetically transmitted. Although cultural *transmission*—in which one individual acquires elements of culture from another—is observed in many species, cultural *evolution* is much rarer (and perhaps, unique to our species). By *evolution*, we mean change that is cumulative (later innovations build on earlier ones), adaptive (new innovations yield some benefit for their bearers), and open-ended (the space of possible innovations is unbounded, since each innovation can give rise to spin-offs).^2^

Formal theories and models of cultural evolution must be able to accommodate discontinuities, because they permeate all branches of culture, including art, science, and technology, as well as economic and political systems (Kuhn, 1962; Wilson, 1973; Bar-Yosef, 1998). They present a formidable challenge for models of cultural evolution, which often have built-in assumptions about how new innovations build on established knowledge (Lewis and Laland, 2012; Montrey and Shultz, 2020). The assumption that new knowledge in a domain builds incrementally on existing knowledge in that domain is inconsistent with findings that entrenchment in established practices and perspectives can hinder innovation, and cultural breakthroughs often come about by striking out in an altogether new direction (Frensch and Sternberg, 1989; Wiley, 1998). For example, if Henry Ford had invested extensive time and energy into perfecting the diet, exercise regime, breeding practices, and so forth, to get a faster horse, he likely would never have invented the automobile. Since cultural change is not necessarily incremental, and not always confined to a given domain, cultural lineages cannot be documented merely through the analysis of cultural outputs. A theory of cultural evolution must incorporate the conceptual networks and psychological processes that spawn cultural innovations. Moreover, such a theory must be non-normative; it must be able to account for individual and group differences in the organizing of knowledge and experience, so as to address how these differences lead individuals and groups to uncharted terrain, and guide their explorations therein, resulting in cultural novelty.

Some models of cultural evolution do an excellent job of revealing patterns of continuity and discontinuity arising through innovative “tweaks and leaps” (Kolodny et al., 2015; Miu et al., 2018). However, their focus is on global patterns (as opposed to underlying mechanisms), and limited to a specific kind of discontinuity that reflects rapid change in the number of elements in a population's toolkit (Kolodny et al., 2015). It is commonly assumed that a cultural discontinuity is due to an “intermediate” that is missing from the archaeological record, but often the discontinuity is due not to a lost intermediate but to complex cognitive processes that are not evident from examination of the cultural outputs themselves (Heyes, 2018a,b; Osiurak and Reynaud, 2020). Such discontinuities may involve modification of superficial structure despite a preservation of deep structure, as occurs in metaphor (Lakoff, 1993), analogy (Gentner, 1983; Holyoak and Thagard, 1996), and cross-domain transfer. In the early scattered stages of cultural evolution research, these discontinuities can be ignored as outliers, but to build a *theoretical framework* for cultural evolution, they must be accommodated. Such a framework requires a process model of how and why cultural lineages unfold as they do, i.e., how a given modification builds upon existing conceptual structure, and paves the way for future conceptual structure.

Recognition of these issues has led some to develop network-based and systems-based models of cultural evolution (Gabora, 1995; Enquist et al., 2011; Kirby, 2017; Gabora and Smith, 2018; Buskell et al., 2019; Tangherlini et al., 2020). By incorporating actual and potential relationships amongst *mental representations* (MRs),^3^ networks provide a means of escaping local maxima (e.g., breeding a faster horse) and finding global maxima (e.g., inventing the automobile). Network-based approaches to culture have also been developed to overcome concerns with phylogenetic approaches (discussed in detail elsewhere, e.g., Lipo, 2005; Gabora, 2006; Tëmkin and Eldredge, 2007; Gabora et al., 2011; Veloz et al., 2012; Gabora and Steel, 2021).

This paper proposes a method for modeling cultural discontinuities caused by cross-domain cognitive processes using Reflextively Autocatalytic Foodset-generated (RAF) networks. The term *reflexively* is used in its mathematical sense, meaning that each part is related to the whole. The term *autocatalytic* will be defined more precisely shortly, but for now it refers to the fact that the whole can be reconstituted through interactions amongst its parts. The term *foodset* refers to the elements that are initially present, as opposed to those that are the products of interactions between them. The psychological significance of this is that the MRs that collectively form a RAF are mutually accessible, i.e., there exists some possible associative path (either direct, or indirect) from any one mental representation in the RAF to any other. Thus, MRs can be compared, contrasted, and strung into sequences, and mental operations, such as addition and subtraction, concept combination, and so forth, are possible.^4^ The study of autocatalytic networks is a branch of network science, the study of complex systems composed of interacting parts. The parts are represented by *nodes*, and interactions amongst them are represented by *edges*. Thus, the nodes of a network are vertices where the edges intersect. In applications of network science to cognition, the nodes generally represent concepts or other sorts of MRs, and the edges represent relationships between them, such as associations due to similarity (e.g., Mars and Jupiter) or complementarity (e.g., mortar and pestle). Autocatalytic networks differ from other network models used to model cognition in that the nodes are not just passive transmitters of activation; they actively galvanize, or “catalyze” the synthesis of novel (“foodset-derived”) nodes from existing ones (the “foodset.”) This makes them uniquely suited to model how new structure grows out of earlier structure, i.e., generative network growth (Steel et al., 2019).

The paper begins with a discussion of cross-domain transfer, followed by an introduction to the autocatalytic approach used to model it using RAF networks, and an overview of past applications of RAF networks to cultural evolution and cognition. We then present a study of a specific cultural discontinuity involving cross-domain transfer of elements of an inspirational source to creative targets. The inspirational source is a celebrated example of Upper Paleolithic art, the Löwenmensch or “lion-man” figurine (from the Hohlenstein-Stadel cave in Germany). The creative outputs are pieces of music, writing, and visual art inspired by this statue. Finally, we present a RAF network model of this cultural discontinuity using an instance of cross-domain transfer.

## 2. Cross-Domain Transfer

Cross-domain transfer is ubiquitous in the history of innovation (Feinstein, 2006; Enkel and Gassmann, 2010; Kalogerakis et al., 2010), and it has been suggested that the capacity to accommodate it could serve as a litmus test for a viable theory of cultural evolution (Gabora, 2019a). The cross-domain source material may arise by noticing something in the environment. For example, the concept of wing warping—the key insight in the invention of the airplane—occurred to Wilbur Wright as he idly twisted an inner tube box (Heppenheimer, 2003). He realized that by twisting the trailing edges of the wings in opposite directions it would be possible to control the direction of the aircraft, thereby removing the remaining fundamental obstacle to human flight.

Cross-domain inspiration is prevalent in the arts. The re-presentation of poetry, painting, or sculpture as music is historically ubiquitous.^5^ A well-known example is Schubert's “Hymn to Mary,” also known as “Ellens dritter Gesang” (“Ellen's Third Song”) or (more famously) “Ave Maria.” This song from the opera *The Lady of the Lake* was inspired by Walter Scott's epic poem of the same title (Deutsch, 1928). Indeed, the phenomenon of cross-domain creativity may go back much further, as the earliest stringed instrument may have been derived from the bow and arrow (Montagu, 2017). In short, cross-domain thinking exerts a profound impact on cultural evolution.

The cross-domain thought that goes into a creative work is not always evident in the work itself. The prevalence of cross-domain influences on creativity was examined in two studies, one with creative experts, and the other with undergraduate students from diverse academic backgrounds (Scotney et al., 2019). Participants listed both their creative outputs, and the influences (sources of inspiration) associated with each of these outputs. In both studies, cross-domain influences on creativity were found to be widespread, and indeed, more frequent than within-domain sources of inspiration. In another study in which painters were instructed to paint what a particular piece of music would “look like” if it were a painting, naïve participants were able to correctly identify at significantly above chance which piece of music inspired which painting (Ranjan et al., 2013). Although the medium of expression was different, something of its essence remained sufficiently intact for people to detect a resemblance between the new creative output and its inspirational source. These studies show that even when the creative output lies squarely in one domain, the process giving rise to it may be rooted in another. A viable theory of cultural evolution must be able to incorporate this.

Cross-domain thinking often takes the form of analogy. Analogy is central to our humanness (Holyoak and Thagard, 1996; Hofstadter and Sander, 2013), and satisfies our need to understand the world through symbolic language (Hart, 2011; Riddell, 2016). It can thus help us in the task of understanding how culture evolves (Brand et al., 2020). A well-known example of analogical transfer is Kekulé's discovery of the structure of benzene by visual analogy to a snake biting its tail (Findlay, 1965; Gabora and Steel, in press). This example reveals an interesting aspect of the phenomenon: the information from the source domain is not always retrieved from memory in the form it was originally encoded. Items from memory may need to be modified to be useful as a source (Holyoak and Thagard, 1996). (Kekulé had likely never seen a snake bite its tail, but his daydreaming mind modified the concept SNAKE in a way that elicited this insight.).

Analogy plays a role not only in the discovery and development of ideas, but in the pedagogical process by which cultural information is transmitted (Holyoak and Thagard, 1996). Analogies are often used by parents and educators to explain abstract, or unfamiliar ideas, in terms of concrete or familiar ones. For example, analogy to existing methods of gene editing played a role in the discovery of CRISPR-Cas9 technology (Doudna and Charpentier, 2014), and its discoverers explain how it works through analogies to a weapons defense system and a Swiss army knife (Doudna and Sternberg, 2017; Thagard, 2019). Thus, analogies influence—sometimes misleadingly—how new concepts are understood, and developed further. Analogy and cross-domain transfer rely on cross-domain associations that often go unnoticed, or arise spontaneously due to the impact of context, and, therefore, cannot be predicted in advance. These associations, in turn, rely on the inter-connectivity of the human brain (Gabora, 2010a). For this reason, it would appear that a model of cultural evolution cannot accurately accommodate lines of cultural influence that arise through these processes unless network structure is at its core.

## 3. The Autocatalytic Approach to Cultural Evolution

Because an RAF captures the nature of a structure *as a whole*, a cognitive RAF is, by necessity, abstract. It does not distinguish between semantic memory (memory of words, concepts, propositions, and world knowledge) and episodic memory (personal experiences); indeed, we are sympathetic to the view that these are not as distinct as once thought (Kwantes, 2005). As in other applications of network science to cognition, in the RAF models developed here, nodes represent MRs. MRs are composed of one or more *concepts:* mental constructs such as DOG or FREEDOM that enable us to interpret new situations in terms of similar previous ones. For a detailed comparison between cognitive RAFs and other models used in cognitive science and cultural evolution research, we refer the reader to papers in which RAFs have previously been used to model the origin of cultural evolution (Gabora and Steel, 2020b, 2021), and the transition to behavioral and cognitive modernity (Gabora and Steel, 2020a). The present paper does not address the *origin* of culture, nor in a general sense how it *evolves*, but it contributes to this overarching program of research by modeling a particularly challenging aspect of cultural evolution, namely, the puzzle of cultural discontinuities.

### 3.1. Rationale for the Approach

We now summarize the key advantages of the RAF approach.

1. **Semantic grounding**. The distinction between foodset items and foodset-derived elements provides a natural means of grounding abstract concepts in direct experiences; foodset-derived elements emerge through interactions, described as “reactions,” that can be traced back to foodset-items.
2. **Reactivity of ideas**. The rationale for treating MRs as catalysts comes in part from the literature on concept combination, which provides extensive evidence that when concepts act as contexts for each other, their meanings change in ways that are often non-trivial and defy classical logic (Osherson and Smith, 1981; Hampton, 1988; Aerts et al., 2009, 2013, 2016). The extent to which one MR modifies the meaning of another is referred to here as its *reactivity*. A given MR's reactivity varies depending on the other MRs present in working memory.^6^ MRs activate, or “react with” each other, by aligning with needs (e.g., the need to solve a problem or resolve cognitive dissonance), and the emotions they elicit. Whereas many conventional node-and-edge network models require external input to continue processing, RAF networks “catalyze” conceptual change endogenously, resulting in new conduits by which needs and goals can be met. The recognition that cognitive networks operate in the service of goals and desires is not new, but here this relationship serves to maintain a reflexively autocatalytic network. By recursively inciting—or, catalyzing—MRs' interactions—or, reactions—between each other, MRs self-organize into new structures that can be formally described. MRs are not only activated by stimuli, and participate in pattern learning, but form a network that is self-organizing and entropy-reducing, and conceptual restructuring can percolate throughout the network and affect its global structure. This is consistent with findings that immersion in a creative task can be therapeutic, and accompanied by a sense of release (Barron, 1963; Forgeard, 2013). Treating MRs as not merely passive participants in spreading activation, but active catalysts of conceptual change is central to our strategy for capturing the flexibility of human cognition. It enables RAFs to be used to model cognitive development during childhood of the kind of conceptual structure that actively participates in the generation of cultural novelty, i.e., the emergence of new “hubs” in the cultural evolution machinery (Gabora et al., 2021).
3. **Tracking cultural change within and across conceptual networks**. The RAF approach tags mental representations with their source, i.e., whether they were (1) innate, (2) acquired through social learning (of *pre-existing* information), (3) acquired through individual learning (of *pre-existing* information), or (4) the result of creative thought (resulting in *new* information). This demarcation makes it possible to trace innovations back to the individuals that generated them, and describe and track how new ideas and cultural outputs emerge from previous ones. We note that in the cultural evolution literature, it is commonly assumed that two processes contribute to cultural evolution: social learning (including iterated learning), and individual learning (Rogers, 1988; Gabora, 1995; Henrich and Boyd, 2002; Mesoudi et al., 2006, 2015; Kirby, 2017).^7^ Creative thought gets lumped in with individual learning, but there is an important distinction between them. In *individual learning*—obtaining pre-existing information from the environment by nonsocial means through direct perception—the information does not change form once the individual knows it. Noticing for oneself that lightning tends to be followed by thunder is an example of individual learning. In contrast, in *abstract thought*—the processing of internally sourced mental contents—the information is in flux (Barsalou, 2005), and when this incremental honing process results in the generation of new and useful or pleasing ideas, behavior, or artifacts, it is said to be *creative* (Basadur, 1995; Feinstein, 2006; Chan and Schunn, 2015a; Gabora, 2017). An example of abstract thought is insight through concept combination, such as by fusing the concept of TIRE with the concept of SWING to yield the idea of making a swing from a tire. Distinguishing between individual learning and creative thought enables us to monitor where in a cultural lineage each new idea (or idea component) first arose, assess the relative contribution of these different sources on the emerging conceptual networks of individuals and social groups, and track cumulative change in cultural lineages step by step.
4. **Integrative framework**. The RAF approach offers an established formal framework for integrating cultural evolution research with cognitive science, and embedding this synthesis in the study of self-organizing structures and their role in evolutionary processes. RAFs replicate and evolve, as demonstrated (both in theory and in simulation studies), which makes them a viable candidate for explaining how cultural information replicates and evolves (Gabora and Steel, 2020b). Indeed, the fact that autocatalytic networks (such as RAFs) have proven useful for modeling the origins of both biological (Vasas et al., 2012; Hordijk and Steel, 2016; Steel et al., 2019; Xavier et al., 2020), and cultural evolution (Gabora, 1998, 2000; Gabora and Steel, 2020a,b, 2021),^8^ suggests that RAF theory may provide a broad conceptual framework that is applicable to the origins and early stages of any evolutionary process.

### 3.2. Reflextively Autocatalytic and Foodset Generated (RAF) Networks

The theory of autocatalytic networks grew out of studies of the statistical properties of *random graphs* consisting of nodes randomly connected by edges (Erdös and Rényi, 1960). As the ratio of edges to nodes increases, the size of the largest cluster increases, and the probability of a phase transition resulting in a single giant connected cluster also increases.

The recognition that connected graphs exhibit phase transitions led to their application to efforts to develop a formal model of the origin of life (OOL), i.e., how abiogenic catalytic molecules crossed the threshold to the kind of collectively self-sustaining, self-replicating, evolving structure we call “alive” (Kauffman, 1986, 1993). In the application of graph theory to the OOL, nodes represent catalytic molecules, and edges represent reactions. It is exceedingly improbable that any catalytic molecule present in the primordial soup of Earth's early atmosphere catalyzed its own formation. However, reactions generate new molecules that catalyze new reactions, and as the variety of molecules increases, the variety of reactions increases faster. As the ratio of reactions to molecules increases, the probability that the system will undergo a phase transition increases. When, for each molecule, there is a catalytic pathway to its formation, the set of molecules is said to be collectively *autocatalytic*, and the process by which this state is achieved has been referred to as *autocatalytic closure* (Kauffman, 1993).

Autocatalytic networks were also used to model how discrete MRs coalesce into an integrated understanding of the world capable of generating cultural novelty (Gabora, 1998).^9^ In this application, nodes represent, not catalytic molecules, but MRs, and the edges represent interactions amongst MRs, such as through associative learning or concept combination. The catalyst often takes the form of a problem, desire, or need. It may be a biological need, such as the need for food or shelter, or produced by some earlier “reaction,” as when solving one problem leads to another. Very often, a reaction on one MR is catalyzed by another MR that has become aligned with the underlying need. For example, although Wilbur Wright's invention of wing warping was ultimately motivated by the need to create a flying machine, it was directly “catalyzed” by the notion of a bending box.

Autocatalytic networks have been developed mathematically in the theory of Reflexively Autocatalytic and Foodset-generated (RAF) networks (Hordijk and Steel, 2016; Steel et al., 2019). The term *reflexively* is used in its mathematical sense to mean that every element is related to the whole. The term *foodset* refers to the reactants that are initially present, as opposed to those that are the products of catalytic reactions. RAF theory has been used to model the emergence of a self-sustaining, self-replicating structure (i.e., a living protocell Hordijk and Steel, 2015). Thus, RAFs offer a promising avenue for modeling the OOL, and thereby understanding how biological evolution began (Vasas et al., 2012; Hordijk and Steel, 2016; Steel et al., 2019; Xavier et al., 2020).

RAFs have also been used to model cognitive transitions underlying cultural evolution (Gabora and Steel, 2020a,b, 2021). This is consistent with the theory that humans possess two levels of complex, adaptive, self-organizing structure—an organismal level and a psychological level with the mind playing the role in cultural evolution that the soma plays in biological evolution (Maturana and Varela, 1973; Barton, 1994; Gabora, 2004). The self-sustaining, self-protecting nature of a conceptual network is evident in the tendency to reduce cognitive dissonance, resolve inconsistencies, and preserve existing schemas in the face of new information. This is not merely an extension of organismal needs; indeed, these two levels of endogenous control can be at odds (e.g., a scientist immersed in solving a problem may neglect offspring or forget to eat.) Although the contents of a conceptual network change over time, it maintains integrity as a relatively coherent whole. Conceptual structure replicates, in a piecemeal manner, when individuals share ideas and perspectives. We posit that the generation of cumulative cultural novelty reflects the capacity for conceptual networks to evolve. Elsewhere, we have extensively compared and contrasted how RAF theory terminology applies in biological and cultural/cognitive settings (Gabora and Steel, 2017, 2020a,b, 2021, in press). The fact that RAFs have proven useful in both these domains suggests that RAF theory may provide a broad conceptual framework that is applicable to the origins and early stages of diverse evolutionary processes.

We now summarize the key concepts of RAF theory. A network of interrelated parts, such as a conceptual network, is referred to as a *catalytic reaction system* (CRS), and is modeled as a tuple Q=(X,R,C,F) consisting of a set *X* of interacting elements, a set R of reactions, a catalysis set *C* indicating which types of elements catalyze which reactions, and a subset *F* of *X* called the foodset. A *Reflexively Autocatalytic and F-generated* set—i.e., an RAF—is a non-empty subset $R^{\prime }\subseteq R$ of reactions that satisfies the following two properties:

1. *Reflexively autocatalytic*: each reaction $r\in R^{\prime }$ is catalyzed by at least one element type that is either produced by $R^{\prime }$ or is present in the foodset, *F*; and
2. *F-generated*: all reactants can be generated from the foodset *F* through a series of reactions in $R^{\prime }$ itself.

Recall that, in a cognitive context, the foodset, refers to those MRs that the individual was born with, or that were obtained through social learning or individual learning; i.e., it excludes MRs resulting from creative mental operations (such as concept combination or induction) in the mind of the individual. Thus, psychologically, the first requirement means that each creative mental operation can be triggered (galvanized) by an MR in the RAF. The second requirement means that, starting from the foodset, there exists a possible stream of thought (series of mental operations) that culminates in each MR in the RAF.

A set of reactions that forms a RAF is simultaneously self-sustaining (by the *F*-generated condition) and (collectively) autocatalytic (by the RA condition) because each of its “reactions” (or interactions) is “catalyzed by” (or facilitated by) an element of the RAF. RAF theory has proven useful for identifying how phase transitions might occur, and at what parameter values. In the origin of life context, an RAF emerges in systems of polymers (molecules consisting of repeated units called monomers) when the complexity of these polymers (as measured by maximum length) reaches a certain threshold (Kauffman, 1993; Mossel and Steel, 2005). The phase transition from no RAF to an RAF incorporating most or all of the molecules depends on (1) the probability of any one polymer catalyzing the reaction by which a given other polymer was formed, and (2) the maximum length (number of monomers) of polymers in the system. This transition has been formalized and analyzed (mathematically, and using simulations) and applied to real biochemical systems (Hordijk and Steel, 2004, 2016; Mossel and Steel, 2005; Hordijk et al., 2010, 2011), as well as cognitive systems (Gabora and Steel, 2020a,b, 2021). Because of the deep structural or algorithmic similarity between the origin of life and the origin of culture (as discussed above), much of this analysis can be readily imported from the former to the latter.

### 3.3. Hierarchical RAF Structure

There are three ways in which subRAFs can combine and expand (Steel et al., 2020):

1. If a CRS (such as a network of catalytic molecules or a conceptual network) contains an RAF, then the collection of all its RAFs forms a partially ordered set (i.e., a poset) under set inclusion, with a unique maximal element: the *maxRAF*. Thus, the maxRAF is the largest RAF in the CRS. A CRS need not have an RAF, but when it does there is a unique maxRAF. Those RAFs that are not the maxRAF are called *subRAFs*. The fact that the maxRAF may contain many RAFs enables RAFs to evolve, as demonstrated (both in theory and in simulation studies) through selective proliferation and drift acting on possible subRAFs of the maxRAF (Vasas et al., 2012; Hordijk and Steel, 2016). The union of any two (or more) subRAFs is an RAF (which explains why there is a unique maximal RAF). There may be a large number of irreducible RAFs, referred to as *irrRAFs*: RAFs that cannot be broken down into smaller RAFs. It is computationally straightforward to determine if a network has a unique irrRAF.
2. Some RAFs have the additional property of being *closed*, meaning that they are stable unless the reaction or foodset changes. Formally, a closed RAF is a RAF that contains every reaction in the network that has each of its reactants and at least one catalyst present either in the foodset or as a product of some reaction in the RAF. The maxRAF is always closed. A subRAF that is not closed is said to be *transient*. A transient RAF may add additional reactions until it becomes closed. The *closure* of any subRAF will contain the original subRAF, and be larger (unless the original subRAF was already closed).
3. *R*′ may combine with a *co-RAF*: a nonempty set of reactions that is not an RAF on its own, but that forms a RAF when combined with *R*′.

These are the only intrinsic processes by which RAFs can expand. Expansion can also be extrinsically driven by a change in the foodset, due to the presence of a new environmental stimulus, or through social learning processes. Extrinsic expansion can also arise due to a change in the permitted reactions, such as when participants in an experiment are instructed to “think creatively.” Computationally, it is straightforward to determine if an RAF is the union of two smaller RAFs, and if a set of reactions is a co-RAF (Smith et al., 2014).

## 4. The Hohlenstein-Stadel Löwenmensch (Lion-man)

**Figure 1:**
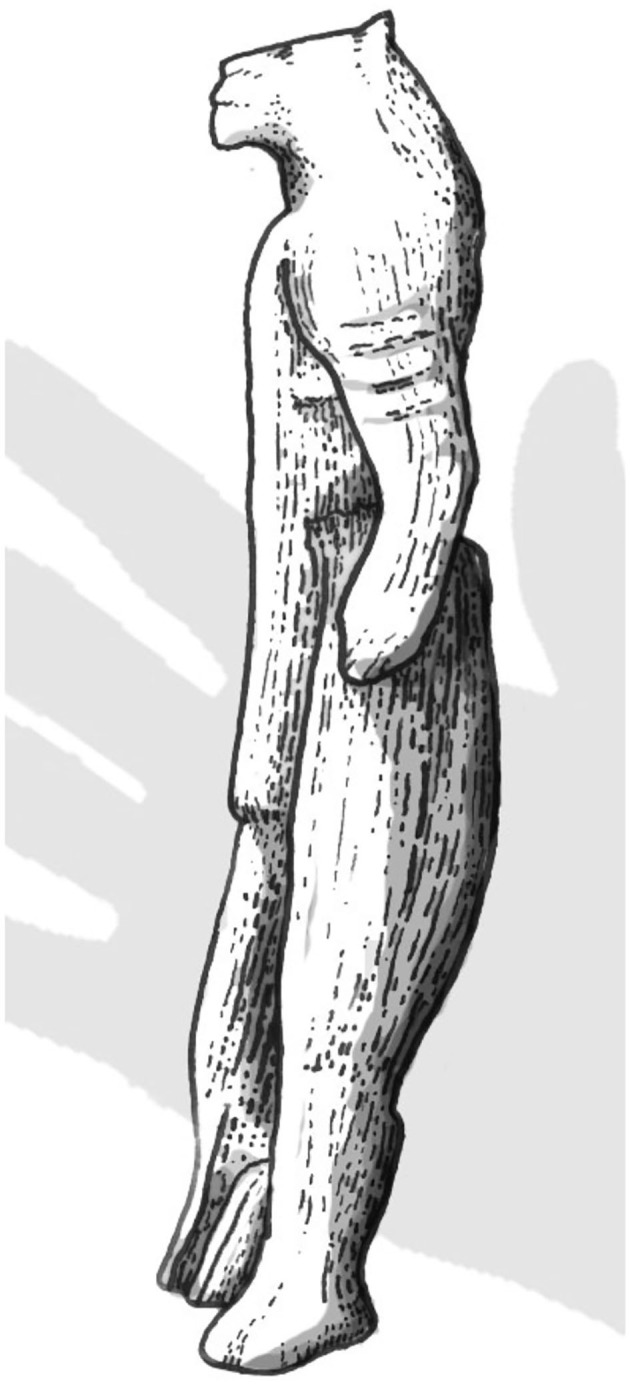
Sketch of the Löwenmensch or “lion-man” figurine from the Hohlenstein-Stadel cave in Germany. According to the Ulm Museum, ^14^C dates put it at an age of 35,000 to 40,000 years. The hand indicates its relative size (Obtained with permission from the artist, Cameron Smith).

The Löwenmensch or “lion-man” figurine from the Hohlenstein-Stadel cave in Germany (Figure 1), carbon-dated to the Interpleniglacial period between 35,000 and 40,000 years ago, is one of the oldest-known zoomorphic (animal-shaped) sculptures in the world, and one of the oldest-known examples of figurative art. It measures 31.1 cm, and was carved out of mammoth ivory using a flint stone knife.

We cannot know exactly how the Löwenmensch figurine was created. However, by reverse-engineering the process, it is possible to infer what conceptual structure would, at a minimum, have had to be in place for it (Tehrani and Riede, 2008; Gabora et al., 2011; Veloz et al., 2012). Our earlier autocatalytic model of the cognitive processes involved in the generation of the Löwenmensch figurine (Gabora and Steel, 2020a) made use of available evidence, such as our knowledge that the lion was the largest and most dangerous predator in the ecosystem of the Interpleniglacial period (Porr, 2010; Kind et al., 2014), and likely a source of fear and awe due to its power and aggression (Hahn, 1986).

## 5. Study

To test the hypothesis that in cross-domain transfer there are identifiable threads of continuity connecting the inspirational source with the output it inspires, which can serve as the basis for lines of cultural descent, we carried out a study of artistic works inspired by the Löwenmensch figurine. We also sought to determine, if such threads of continuity exist, what form they take.

### 5.1. Participants

The researchers contacted musicians, visual artists, and composers with whom LG was familiar, to ask them if they would like to participate in the study, i.e., convenience sampling. Participants were recruited by KG through email. This resulted in seven participants/participant groups, consisting of one musician, one musical group (consisting of three members), one composer, one visual artist, one poet, and two writers. Demographic information about the participants is provided in Table 1.

**Table 1:** Demographic information.

**Artist**	**Primary medium**	**Age**	**Nationality**	**Years of experience**
SLT^*^				
AS	Music - Percussion	33	USA	> 15
LN	Music - Vocals	36	Canada	> 20
ND	Music - Cello / Computer	27	Persia/Russia	> 10
SW^*^	Written word (Novelist)	71	Australia	> 30
WC^*^	Music	67	Australia	> 30
MG	Music	38	USA	> 20
LH	Written word (Poetry)	78	Australia	> 30
SD	Visual Art	64	Canada/USA	> 30
SB	Written word (Novelist and Playwright)	67	USA	> 30

* *Indicates participants who provided process descriptions (SLT refers to the musical group composed of the three individuals, AS, LN, and ND)*.

### 5.2. Method

Upon obtaining verbal consent, KG emailed the participants a pamphlet about the Löwenmensch figurine, including its physical description, history, and context of discovery (available in the Supplementary Information). Participants were instructed to read the pamphlet, view the picture of the figurine, and use it as an inspiration for a creative product. They were also asked to document their process of thinking and creation, and answer five questions that were emailed to them:

1. What were your initial reactions to the figurine? What were your thoughts and emotions when you first encountered the object and its history, in the context of a creative prompt to compose music/poetry?
2. How did your idea for your creative product emerge? What were some of the initial ideas that came up, and was there any specific rationale for choosing the ideas that you did?
3. What were your thoughts and emotions as you were engaged in the process of creation? What was the process itself like?
4. What were your thoughts and emotions when your creation was complete?
5. Has your creative involvement with the figurine triggered other ideas and/or affected other areas in your life?

The finished artworks inspired by the Löwenmensch figurine are accessible in the Supplementary Data section of the paper. As such, they are able to exert an influence on the cultural lineage of scholarly inquiry into the nature of cultural evolution and the creative processes that fuel it. However, the influence of these Löwenmensch-inspired artworks is not only scholarly in nature, since some of the musicians made their pieces of music available on the internet and a live performance of the music written by the musicians has been planned for the spring of 2022. In some cases, the musicians' websites also included information about the figurine itself, thereby expanding the scope of influence of the figurine itself, and its artistic cultural “offspring.”

### 5.3. Data

Seven creative pieces in the form of images, poetry, and music were submitted by participants. Although we asked all participants to provide responses to the questions, we only received responses from two individual participants and the musical group, which were used in the analysis below. The participants' creative works are available in the Supplementary Information.

### 5.4. Analysis and Results

To analyze the data, we used a qualitative data analysis method known as thematic analysis (Braun and Clarke, 2012). Each of the participants' descriptions of their creative process was read over several times by KG, and specific extracts of text were assigned codes. For example, the extract, “We really wanted to focus on the fact that the artist chose a lion and a human” was coded “lion-human hybrid.” The initial codes were then refined and organized into themes. A theme could pertain to any aspect of the figurine, its history, or context, that inspired one or more elements of the participants' creative output. The assignment of codes and the grouping of codes into themes facilitated the process of identifying commonalities across the data provided by the different groups.

The presence or absence of a specific theme in a process description is depicted in Table 2. Three themes appeared multiple times, i.e., in at least two of the three creative process descriptions: Lion-Human hybrid (LH), Deterioration / erosion by natural forces (D), and Waiting to be found (with a story to tell) (W). These three themes therefore served as three of our four through-lines. The fourth through-line, Subtractive sculpting (S), appeared only in SLT's description. We nevertheless retained it as a through-line because it was not straightforwardly implementable in any domain other than music, and SLT emphasized its central importance to their creative output. Therefore, it was deemed to be of greater significance than any other theme that appeared only once.

**Table 2:** Presence (+) or absence (-) of through-line in the work of the creators.

	**Musical group (SLT)**	**Writer (SW)**	**Musician (WC)**
Lion-human hybrid	+	+	+
Subtractive sculpting / negative space	+	-	-
Deterioration / erosion by natural forces	+	+	-
Waiting to be found (with a story to tell)	-	+	+

We then reviewed the creative output to confirm that each of the four identified through-lines had manifested in at least one of the creative outputs themselves, and this was indeed the case (e.g., the short-story described a lion-human hybrid, and subtractive sculpting was evident in the music, which began with a chaotic blend of many sounds, many of which had been “whittled away” by the end to produce a sound that was purer and more musical). We now elaborate briefly on the four through-lines:

1. Lion-Human hybrid (*LH*): All three creative process descriptions indicated how the anthropomorphic nature of the figurine was an important source of inspiration. For the group of musicians, this manifested as the fusing of the sound of a lion roar with more “human” elements (i.e., instrumentation). For writer SW, it manifested as a story describing the deep bond between a misunderstood girl and her friendship with a lion. For composer WC, this “humanoid” nature of figurine led to exploring possibilities of the figurine being one possibly used for ceremonial protection, making the creative product a piece of music meant for a chorus of individuals. As such, the creators attempted to capture the fusing of human and animal in their creative pieces.
2. Subtractive sculpting/negative space (*S*): Subtractive sculpting is the action of *removal* of material from a whole until a desired form is reached. The musical group (SLT) took inspiration from this to attempt to model their process after the action of subtractive sculpting. They began with a “block” of sound, and proceeded to remove notes from the same to arrive at a finished product. They noted that this was counter-intuitive to the typical method of creating music—“Instead of silence being the ‘medium’ and sound the ‘paint,’ the opposite had to occur in order for a true analogy to be made to the process of carving.” Similarly, one of the members of SLT created a piece of art inspired by negative space—a relief drawing of the roots of a now absent tree through tracing the negative space left by it in the soil.
3. Deterioration/Erosion by natural forces (*D*): The creators described being inspired by the figurine's age. SW described imagining holding the figurine, and tracing its surface to capture the wear-and-tear it must have experienced over thousands of years. WC indicated how her piece of music was to this reconstruction of the figurine in the present day, rather then what it had been when it was undamaged. SLT attempted to capture through music how the figurine went from being something that was likely frequently held and cherished, to something that was “abandoned” to the elements. The piece begins slowly, reaches a crescendo, and then fades. In their words, “We anticipate that what we created as our anthropomorphic sound sculpture will deteriorate slowly like the Lion-Man sculpture has over 40,000 years.” In contrast, WC imagined this wear-and-tear as the figurine as having “shattered” over time due to “despair.”
4. Waiting to be found (with a story to tell) (*W*): Participants described being inspired by the discovery of the figurine. The context of its excavation and subsequent reconstruction led some creators to imagine that the time had come for the figurine to tell its story to the world. SW described feeling a “growing conviction” that she “knew the true story of the figurine,” and chose to write her piece in the first person, as the figurine narrating its own life to the reader. Upon completing her piece, she described experiencing a deep sense of “relief,” and felt as if she had “done right by the figurine.”

Thus, we identified four through-lines by which characteristics of the figurine manifested in the creative work: (1) the notion of a lion-human hybrid, abbreviated *LH*, (2) the notion of negative space, or subtracting from the whole to reveal the form within, which we referred to as subtractive sculpting, abbreviated *S*, (3) the notion of erosion over time by natural forces, or deterioration, abbreviated *D*, and (4) the notion of waiting to be a found with a story to tell, abbreviated (W).

These through-lines, and examples of the evidence upon which they were identified, are provided in Table 3, and the full set of evidence is provided in the Supplementary Materials. These four spontaneously-generated threads of cultural continuity formed the backbone of a Löwenmensch-inspired cultural lineage, enabling cultural evolution even in the face of discontinuity at the level of conventional categories or domains.

**Table 3:** ^*^These are a sample from notes made by participants.

**Cross-domain elements**	**Symbol**	Extract from participants' notes^*^	**Observed in creative output**
Lion-Human hybrid	LH	Our human metamorphosis of the lion's roar is inspired by the anthropomorphic nature of this particular statue, as our ‘humanness’ becomes intertwined with the ‘animalness’ of the lion.	The background of the piece is the sonically elongated sound of a lion roar; It begins with the pure sound of a lion, and human interaction in the form of instrumentation and rhythm is gradually introduced to interact with this sound.
Subtractive sculpting/negative space	S	This *[the audio of the lion roar]* will be the ‘raw material’ that we work with and will represent the sonic equivalent of an uncarved block, bone, or tusk… As musicians, our canvas is generally silence and we tend to add sound to a blank canvas. In this new form of creation, our canvas is instead sound itself, from which we subtract a variety of sonic material.	The piece begins with a full-bodied sound i.e., a lion roar, and degrades over the course of the piece.
Deterioration/erosion by natural forces	D	We anticipate that what we created as our anthropomorphic sound sculpture will deteriorate slowly like the Lion-Man sculpture has over 40,000 years.	
		I found myself suggesting that they *[her students]* imagine running their hands over the figurine… feeling how smooth the ivory had been worn.	For generations, I was cradled in many worshiping hands.
Waiting (with a story to tell) to be found	W	I didn't feel as if I was making anything up, just reporting on what really happened.	“… hid me deep in a cave, in the hope that one day, what I'd become would found a new cult of outsiders who believe in art… for forty thousand years I have waited…”

*The complete list of all instances can be found in the Supplementary Information*.

* *Indicates participants who provided process descriptions (SLT refers to the musical group composed of the three individuals, AS, LN, and ND)*.

In short, identifiable lines of inspiration connected the original creative product (the Lion-man) to novel creations by participants. This process of cross-domain transfer, from sculpture to music, visual art, prose, and poetry is next modeled using RAFs.

## 6. Model

**Figure 2:**
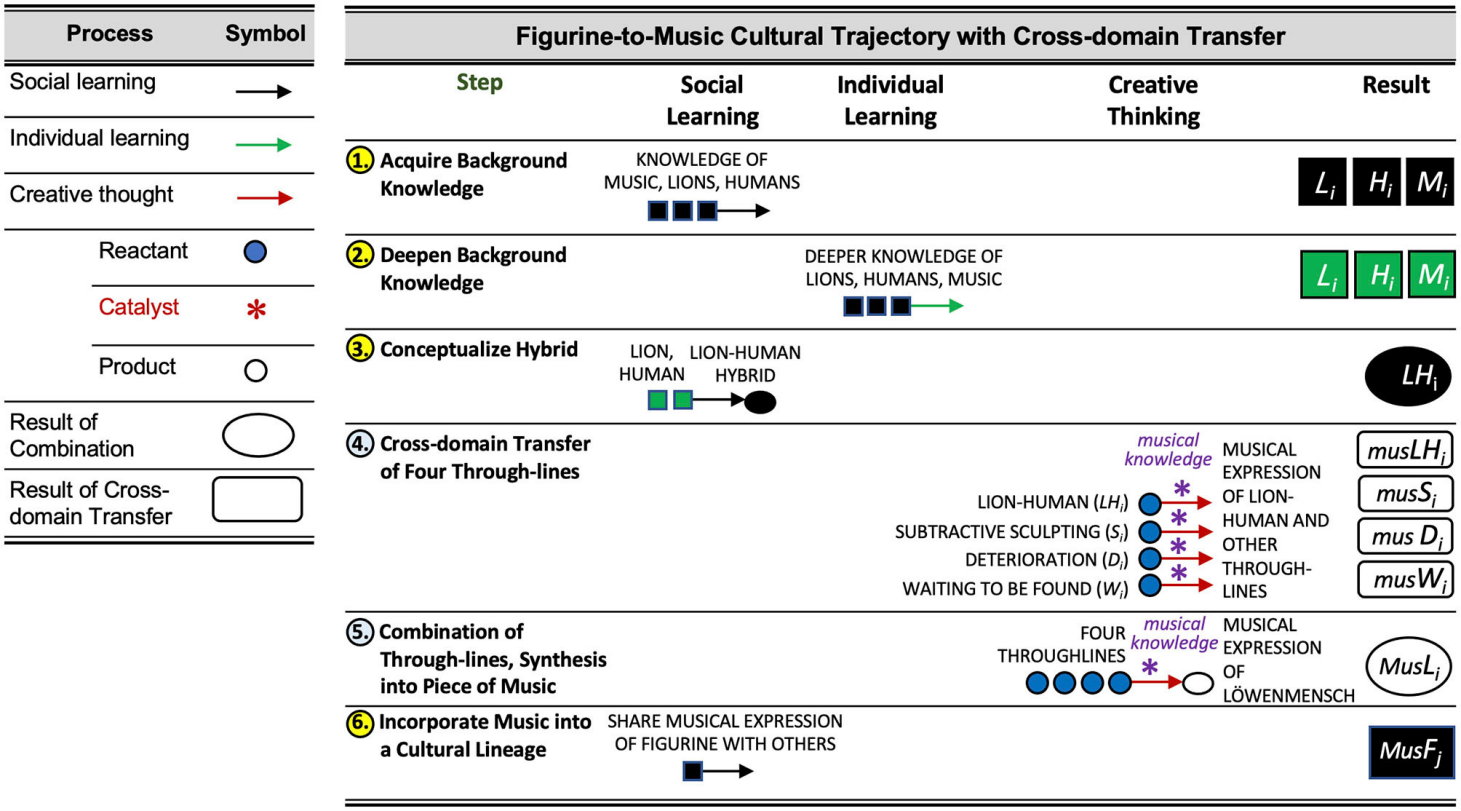
(Left) Processes involved, and symbols used to depict them. (Right) Cognitive steps culminating in the cultural lineage described in the text. Items in black are transmitted through social learning. Items in green are the result of individual learning. Each instance of social learning (Column 2) has been preceded by some individual at some point in the lineage by a relevant instance of individual learning (Column 3) or creative thinking (Column 4). Steps one, three, and six transmit foodset (existing) knowledge amongst individuals. Step two occurs through assimilation of the environment not mediated by social learning. Steps four and five generate new foodset-derived knowledge in the mind of the musician.

**Figure 3:**
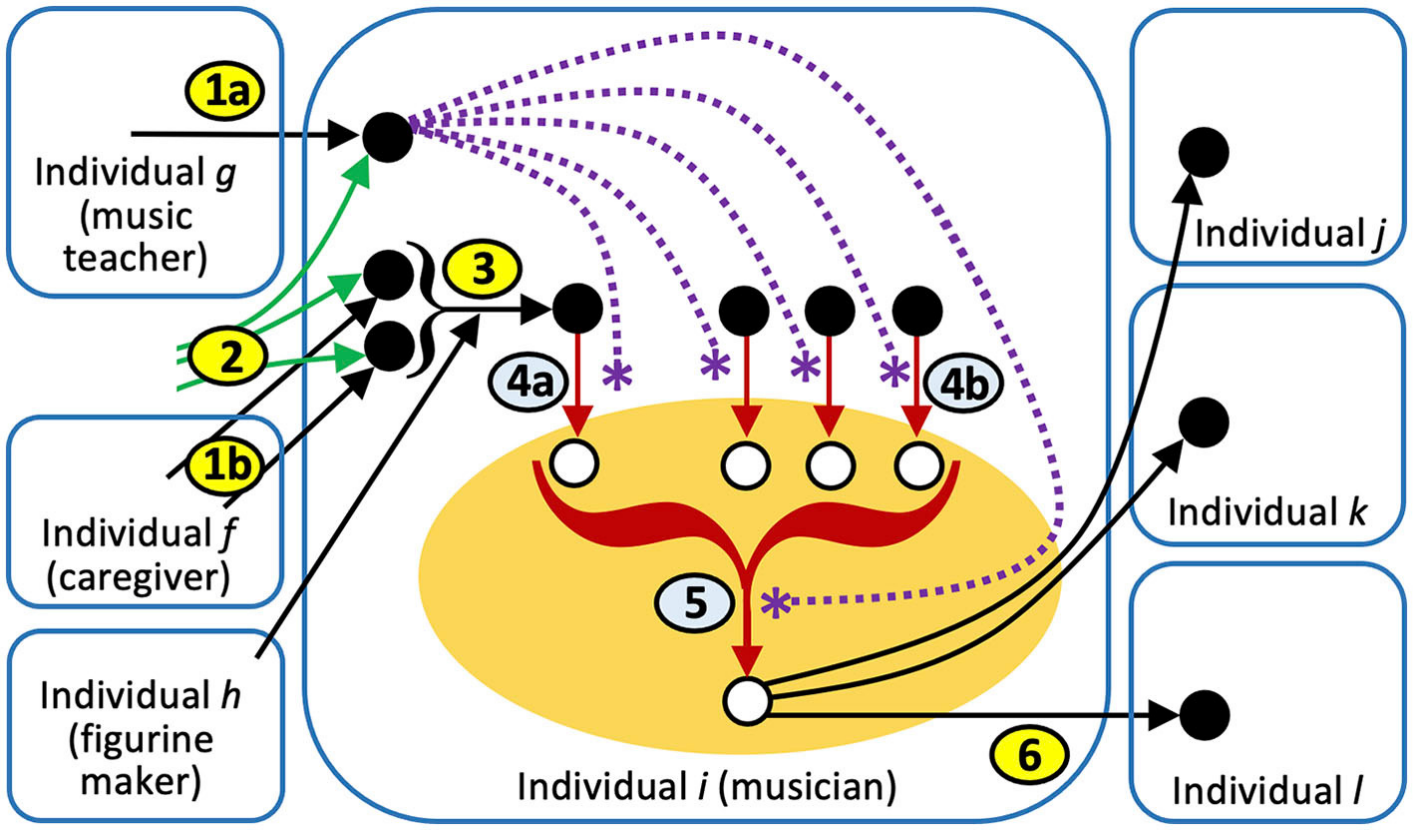
Steps in the cross-domain transfer from figurine to music (These are the same six steps as in the previous figure; see text for details). Black arrows indicate social learning, green arrows indicate individual learning, red arrows indicate creative thought, dotted purple lines with stars indicate “catalysis” of a creative thought. Foodset items are black, and foodset-derived items are white. Since a particular concept, such as the concept of a lion-man hybrid, play different roles in different steps, it is not possible to use color and shapes to depict roles, as in Figure 2. Orange oval represents the domain of music. Purple arrows in steps four and five represent “catalysis,” i.e., the facilitation of a creative thought that would otherwise be unlikely or difficult. These steps are catalyzed by musical knowledge, and the desire to express the experience of the figurine through the constraints of music. Note that since individual *i* obtained the products of step 5 through creative thought, it is not member a of the food-set in *i*, but it is a members of the food-set item in *j*, *k*, and *l*, because they obtained it from *i* through social learning.

We now present an RAF model of the process by which the Löwenmensch figurine inspired these artistic works. The process is summarized in Figure 2, and depicted in Figure 3.

### 6.1. Step One: Socially Learned Background Knowledge

Each creator's output was preceded by a lengthy period sometimes referred to in the creativity literature as *preparation*, which includes the acquisition of domain-specific knowledge. In the case of the musicians, this includes learning to hear and play or sing music by others, and knowledge of a particular instrument, as well as music theory. Recall that *F* refers to the foodset, thus *F*_*i*_ refers to the foodset of individual *i*, the musician, and *S*_*i*_[*M*_*g*_] refers to information acquired by *i* through social transmission from *M*_*g*_. The transmission of knowledge of music from a music teacher, *M*_*g*_, to the young musician, *M*_*i*_ is described as follows:


(1) $$\begin{array}{l} F_{i}\mapsto F_{i}\cup \left\{M_{i}\right\},\;M_{i}\in {𝕊}_{i}\left[M_{g}\right]. \end{array}$$

The musicians and other creators also made use of basic knowledge of lions and humans the was originally obtained by way of caregivers, which would be modeled in the same way as domain-specific (e.g., musical) knowledge.

### 6.2. Step Two: Building on Social Learning

The musicians built on their socially learnt knowledge of music through individual learning experiences involving practice and experimentation. 𝕊_*i*_[*l*] refers to information acquired by *i* through individual learning. Thus, we represent knowledge of music acquired by the musicians through individual learning experiences as follows:


(2) $$\begin{array}{l} F_{i}\mapsto F_{i}\cup \left\{M_{i}\right\},\;M_{i}\in {𝕊}_{i}\left[l\right]. \end{array}$$

The creators also used individual learning to build on their socially learnt knowledge of lions and humans, and that mathematical description of this would be described analogously.

### 6.3. Step Three: Identify Through-Lines

Creative honing is often (though not always) stimulated by *problem finding* (Abdulla et al., 2020), which begins with a sense of uncertainty, incompletion, inconsistency, or need, accompanied by a lingering feeling that compels the exploration and expression of ideas (Feinstein, 2006). In this case, the uncertainty concerned how to express the figurine (including the emotions it elicits) in the creators' domain. In the case of the musicians, this entailed uncertainty as to how to adapt characteristics of the figurine to the new domain of music.

Recall that our participants identified four through-lines, which we refer to as lion-human hybrid (*LH*), subtractive sculpting (*S*), deterioration (*D*), and waiting to be found (*W*). These through-lines undergo cross-domain transfer into the creators' respective artistic domains. Let us continue to focus on the musicians, and model social transmission of the *LH* (lion-human hybrid) through-line from the individual *h* who carved the figurine so long ago, to a musician, individual *i*, by way of our pamphlet about the figurine. The participants had knowledge of lions and humans prior the task, but the Löwenmensch figurine showed them that these concepts could be combined to give a lion-human hybrid. We represent the social transmission of the combination LION-HUMAN from the carver *h*, to the musician, individual *i*, as follows:


(3) $$\begin{array}{l} F_{i}\mapsto F_{i}\cup \left\{LH_{i}\right\},\;LH_{i}\in {𝕊}_{i}\left[LH_{h}\right]. \end{array}$$

Assimilation of the *S*, *D*, and *W* through-lines are modeled analogously.

### 6.4. Steps Four: Cross-Domain Transfer

Let us consider the process by which the musicians carried out cross-domain transfer of the lion-human hybrid (*LH*) through-line resulting in a musical expression of the lion-human hybrid. This kind of mental operation is sometimes referred to as representational redescription, and in the language of autocatalytic networks, it is sometimes called a “reaction.” It transforms an element of *F*_*k*_ into an element of ¬*F*_*k*_. In the case of the musicians and the *LH* through-line, the concept of a lion-human hybrid (*LH*_*i*_) served (in the language of autocatalytic networks) as a “reactant” that undergoes further transformation. The process was facilitated (or, in the language of autocatalytic networks, “catalyzed”) by *musical knowledge*, as well as by the context: *desire to express figurine as music*, which we collectively denote *n*_*i*_. We refer to this kind of need, or knowledge, or desire, that sparks or enables a particular mental operation to take place, as a “catalyst” of that mental operation. Cross-domain transfer generates a *product*, denoted *R*_*i*_ ∈ *musLH*_*i*_. Thus, in general, for an individual *k* (where *k* = *i, j*), we write $a_{k}\overset{b_{k}}{\to }c_{k}$ to denote the process that transforms one MR (*a*_*k*_) to a resulting MR *c*_*k*_ (in ¬*F*_*k*_) by catalyst *b*_*k*_, and we let $a_{k}+a_{k}^{\prime }\overset{b_{k}}{\to }c_{k}$ denote a mental operation that combines and transforms two MRs ($a_{k},a_{k}^{\prime })$ into a new MR *c*_*k*_ (in ¬*F*_*k*_) by catalyst *b*_*k*_. We describe the musicians' cross-domain transfer of the *LH* through-line as follows:


(4) $$\begin{array}{l} LH_{i}\overset{n_{i}}{\to }musLH_{i},\;\neg F_{i}\mapsto \neg F_{i}\cup \left\{musLH_{i}\right\}, \end{array}$$

Cross-domain transfer of the other through-lines, specifically Subtractive sculpting (*S*_*i*_), Deterioration (*D*_*i*_), and Waiting to be found (*W*_*i*_), are described analogously. Cross-domain transfer expands the affordances of MUSIC (i.e., it is now perceived as something that can be express the emotional response to an ancient figurine). Affordances are a kind of association and, as such, they increase the connectivity of the conceptual network.

### 6.5. Step Five: Combining Through-Lines and Synthesis Into Creative Output

Once again, the products of the previous “reaction,” i.e., the musical forms of the through-lines—lion-human hybrid (*musLH*_*i*_), subtractive sculpting (*musS*_*i*_), deterioration (*musD*_*i*_), and waiting to be found (*musW*_*i*_)—serve as “reactants” that undergo transformation through mental operations to achieve the final finished product, a piece of music that conveys the experience of encountering the Löwenmensch. This product is denoted *musL*_*i*_ ∈ 𝕊_*i*_(*l*). This step, like the preceding one, was facilitated, or “catalyzed” by *musical knowledge*, as well as by the context: *desire to express figurine as music*, which we collectively denote *n*_*i*_. We describe this step as follows:


(5) $$\begin{array}{l} musLH_{i}\;+\;musS_{i}\;+\;musD_{i}\;+\;musW_{i}\overset{n_{i}}{\to }musL_{i},\;\neg F_{i}\\ \;\mapsto \neg F_{i}\cup \left\{musL_{i}\right\}, \end{array}$$

The new connections the creators acquired through the process of transferring elements of the figurine from the domain of sculpture to their chosen domain of artistic expression reconfigured their conceptual networks. For example, the realization that the concept LION can be hybridized with the concept HUMAN, and that this new hybrid concept can be re-expressed as music, forged new connections in their conceptual networks. The initial creative phase was followed by a *honing and verification* stage: development of the idea, as well as evaluation and assessment. This involved practicing and improving upon the original musical ideas. The thought processes underlying these activities brought about further expansion of the maxRAF.

### 6.6. Step Six: Incorporating Musical Expression of Löwenmensch Into Cultural Lineage

Once the artist was confident he/she had achieved a musical expression of the figurine, the next stage was to share it with others, for example, by making it publicly available on a website. We represent the social transmission of a piece of music inspired by the figurine denoted *musF*, from a musician, individual *i*, to a listener, individual *j*, as follows:


(6) $$\begin{array}{l} F_{j}\mapsto F_{j}\cup \left\{musF_{j}\right\},\;musF_{j}\in {𝕊}_{j}\left[musF_{i}\right]. \end{array}$$

Note that in the RAF approach, the impact of entire experience (including both the experience of acquiring knowledge about the figurine and the experience of listening to the music) on the listener is described differently for the musicians' audiences than for the musicians themselves. For example, the mental representation of a lion-human re-expressed as music, i.e., *musLH* was obtained by the musician through representational redescription, modeled as a “reaction” that expanded the set of foodset-derived MRs, ¬*F*. In contrast, for the musicians' audiences, *musLH* is obtained as through social learning, and it expands the foodset, *F*. Not only did the creators' insights over the course of the project impact the scope of what is possible in their chosen creative outlet, but it impacted humanity's conception of the figurine as well, by expanding the breadth of associations to include musical, literary, and two-dimensional outputs.

## 7. Discussion and Conclusion

The challenge of cultural discontinuities is sometimes swept under the rug by discarding outliers with characteristics that do not fit neatly into pre-established categories. However, this rules out *a priori* exactly those elements of cultural lineages that have the capacity to open up new cultural trajectories. Such analyses appear to support phylogenetic tree models of culture, but they disqualify those data that have the greatest impact on how culture evolved, data that would make cultural lineages more network-like than tree-like. To describe and explain cultural lineages—including their discontinuities—with as much rigor as has been carried out for biological lineages, we need a theory that incorporates individual and group differences in the structuring of knowledge and experience that give rise to patterns of cultural descent that stray from established classifications. A given song, or tool, or artwork is not just a variant of its predecessors; a host of subtler cultural influences may affect its form and cultural impact, i.e., the feelings it evokes, and the utility people gain from it. This paper investigated the hypothesis that, with respect to one widespread source of cultural discontinuity—cross-domain transfer—threads of cultural continuity can be found, though they do not reflect categorical or domain-based relationships, and cannot be predicted in advance since they arise spontaneously through interactions between the inspirational source, the target domain, and the creator. We identified four such through-lines in the generation of creative works inspired by the Hohlenstein-Stadel Löwenmensch figurine, and used one of them to illustrate how such lines of cultural relatedness can be modeled using the autocatalytic network approach. The RAF approach enabled us to track trajectories of conceptual change within and across individuals, and ultimately understand how these processes culminated in the unique contributions of individuals and groups to cultural evolution. Thus, the approach was well-suited to modeling how new ideas grew out of existing knowledge.

A limitation of this study was the small sample size. In addition, the creative products may have been affected by the limited time frame the creators had to produce their work. Also, we were not able us to assess precisely which aspects of the inspirational input inspired the through-lines; for example, it is uncertain to what extent the through-lines emerged due to visual inspection of the photograph of the Löwenmensch figurine, or to the written document about it that they were given. In future work, the approach will be used to analyze vastly larger cognitive networks, capitalizing on its merits relative to other methods for analyzing large networks, and effectiveness for developing efficient (polynomial-time) algorithms for questions that are computationally intractable (NP-hard) (Steel et al., 2019). Another interesting direction for future work in this area would be to explore the impact of a creative source across other disciplines. For instance, the Löwenmensch figurine may “catalyze”—either directly or indirectly—thinking in disciplines such as archaeology or soil chemistry, or enter into political discussion of the ethical considerations in the use of ivory.

Like other network approaches to culture such as that of Enquist et al. (2011), RAFs model the cognitive structures and processes that influence cultural trajectories. Another complementary albeit non-RAF approach is the work of Carignani et al. (2019). They develop a “Woesian” (after Carl Woese) model of technological discontinuities that they apply to detailed analyses of the invention of the bow-and arrow, the turbojet revolution, and the 3D printer. Unlike other network approaches, the RAF approach differentiates between (1) foodset MRs (knowledge that is either innate, or that results from social learning or individual learning of *existing* information), and (2) non-foodset MRs (*new* information that results from abstract thought, or interactions amongst other MRs). This makes it straightforward to identify the point of origin of each new feature of a cultural lineage, and what prompted or inspired that new feature, as well as the cognitive processes involved. Individual differences in reliance on foodset versus foodset-derived information sources may culminate in different kinds of conceptual networks, learning strategies, or personality dynamics.

The RAF framework predicts that individuals whose conceptual networks contain roughly the same *concepts* and *associations*—i.e., they would be described similarly using standard network approaches—may nevertheless think and behave quite differently, due to different needs and desires (and different degrees to which knowledge has been assimilated into the network as a whole) which may “catalyze” different thought processes. This in turn could result in markedly different cultural contributions. This expected difference is modeled in the RAF approach by describing interactions between MRs as “catalyzed reactions.” In addition, individual differences in the relative reliance on foodset versus foodset-derived knowledge are expected to result in differences in the number, kind, and impact of cultural contributions. For example, while Mozart and Salieri had similar environments, experiences, and social circles, their compositions and cultural impact were markedly different. The RAF approach enables us to capture such distinctions by providing a means of formalizing the notion that cognitive growth may be sparked by cognitive dissonance or a sense that something is unresolved, which triggers the restructuring or redescription of representations, modeled as “reactions.” By incorporating “catalysis,” RAFs can model how new representations form and change when people look at existing ideas from new perspectives, or view known concepts from different contexts, or combine them. We suggest that the difference between Mozart and Salieri reflects, not differences in their knowledge of music and understanding of the rules of composition, but in their proclivity to access life experiences and emotions, mull them over, and allow them to serve as reactants and catalysts in the musical domain. We further suggest that differences in reactivity may help explain why intelligence is a necessary but not sufficient condition for creativity (Jauk et al., 2013).

An RAF-based theory of cultural evolution predicts that productive collaborations require not just overlapping yet different conceptual structures (so that each individual contributes something to the whole). There is some support for this. It has been suggested that creative breakthroughs are more likely to arise from conceptually distant sources than from conceptually close ones (Poze, 1983; Holyoak and Thagard, 1996; Gentner and Markman, 1997; Ward, 1998). There is evidence that individuals from different (often adjacent) fields produce the most creative solutions (Wiley, 1998; Jeppesen and Lakhani, 2010; Franke et al., 2014). Some studies have also shown an advantage of conceptually distant sources over near ones with respect to novelty, quality, and flexibility of ideation (Dahl and Moreau, 2002; Hender et al., 2002; Chan et al., 2011; Chiu and Shu, 2012; Goncalves et al., 2013), while other studies concluded that there was no such advantage (Dunbar, 1997; Chan and Schunn, 2015b), or were inconclusive (Malaga, 2000; Enkel and Gassmann, 2010). Despite their potential benefit, distant sources may be harder to find, and require more iterative processing (Chan and Schunn, 2015b). A related prediction derived from the RAF approach is that productive collaborations require not just overlapping yet different conceptual structures, but compatible levels of MR reactivity. If their joint reactivity is too low, new ideas fail to emerge, but if it is too high, they lose the thread of continuity necessary for cumulative change.

RAF networks have been used to model the origins of evolutionary processes, biological (the origin of life) as well as cultural (the origin of cumulative innovation). We think this is not coincidental; indeed, elsewhere, we showed that both the evolution of early life and cultural evolution are instantiations of a primitive form of evolution—i.e., cumulative, adaptive, open-ended change—referred to as Self-Other Reorganization (SOR) (Gabora, 2019b). Instead of replication using a self-assembly code, SOR entails internal self-organizing and self-maintaining processes within entities, as well as interaction between entities. The argument for SOR bolsters the argument for applying RAFs in both domains, though the viability of the project of describing cultural evolution using RAFs does not hinge on this argument, and places the underlying cognitive processes center stage.

In sum, this work shows that it is possible to incorporate cultural discontinuities into the modeling of a cultural lineage through the identification of spontaneously-generated through-lines, and this has implications for in what sense culture evolves. Using RAF networks to model such cross-domain transfer allows us to better understand the minds of creators, through a systematic mapping of the process of creative inspiration, and its impact on expressions of culture.

## Data Availability Statement

The datasets presented in this study can be found in online repositories. The names of the repository/repositories and accession number(s) can be found in the article/Supplementary Material.

## Ethics Statement

The studies involving human participants were reviewed and approved by UBC Okanagan Behavioral Research and Ethics Board. The participants provided their informed consent to participate in this study.

## Author Contributions

LG conceived of the paper, study, and model and did most of the work on the model. KG did most of the work on the study. They wrote the paper together.

## Conflict of Interest

The authors declare that the research was conducted in the absence of any commercial or financial relationships that could be construed as a potential conflict of interest.

## Publisher's Note

All claims expressed in this article are solely those of the authors and do not necessarily represent those of their affiliated organizations, or those of the publisher, the editors and the reviewers. Any product that may be evaluated in this article, or claim that may be made by its manufacturer, is not guaranteed or endorsed by the publisher.

## Footnotes

^1^An existence proof shows merely that it is possible for something to exist, and as such it requires just a single instance.

^2^The term “cultural evolution” is sometimes used in a less restricted sense to refer to novelty generation and transmission without the requirement of cumulative, adaptive, open-ended change, e.g., Whiten (2019). Non-human animals are capable of limited cumulative cultural change in the sense of improvement with respect to efficiency or number of traits, but as pointed out by Mesoudi and Thornton (2018), humans appear to be uniquely capable of recombining traits and adapting them to new contexts (Gabora, 2019a), cultural exaptation (La Porta and Zapperi, 2020), and niche construction (Odling Smee et al., 2003), which can result in discontinuities.

^3^Although we use the terms “mental representation,” we are sympathetic with the view (common amongst ecological psychologists and in the quantum cognition community) that what we call mental representations do not “represent,” but act as contextually elicited bridges between the mind and the world.

^4^For further details see Gabora and Steel (2017, 2020a,b, in press). Elsewhere, we investigate the relationship between RAF structure and psychological integration/fragmentation [Ganesh, K., and Gabora, L. (under review), A Dynamical Autocatalytic Model of Therapeutic Change].

^5^See, for example, research on ekphrasis, wherein a visual work of art is either verbal described, or translated into some other artistic medium (Goehr, 2010).

^6^For example, the degree to which PYLON qualifies as an instance of HAT increases in the context “worn to be funny” (Veloz et al., 2011).

^7^Much has been made of Rogers' paradox (that social learning can invade a population without increasing its fitness). However, the apparent “paradox” is an artifact of assumptions built into Rogers simulation (Gabora and Tseng, 2017; Kharratzadeh et al., 2017), including that it incorporates social learning and individual learning (of existing information) but not the creative generation of new information.

^8^For related approaches, see Cabell and Valsiner (2013), Muthukrishna et al. (2018), and Andersson and Törnberg (2019).

^9^Autocatalytic networks provide an abstract formal framework that happened to be first applied to the origin of life, but are equally applicable to cognition. Neither is more or less “metaphorical” than the other; they are simply different applications of the same formal framework.
