# Supplementary material for: Modeling Discontinuous Cultural Evolution: The Impact of Cross-Domain Transfer
Source: Front Psychol. 2022 Feb 24;13:786072. doi: 10.3389/fpsyg.2022.786072 (PMC8908956; doi:10.3389/fpsyg.2022.786072)
Supplement: Supplementary file 1 [file Data_Sheet_1.pdf]

Supplementary Information for 'How Cross-domain Inspiration Impacts Cultural Evolution'

Kirthana Ganesh [1] **and Liane Gabora** [2,*]

University of British Columbia

Correspondence*:
Liane Gabora
Department of Psychology, Fipke Centre for Innovative Research, 3247 University Way, University of British Columbia, Okanagan Campus, Kelowna, Canada, V1V 1V7, liane.gabora@ubc.ca

1 INFORMATION ABOUT THE LÖWENMENSCH FIGURINE PROVIDED TO ARTISTS

A pamphlet with information about the discovery, description, and significance of the Löwenmensch (Lion Man) figurine was sent to all participants as an attachment to an email message (Figures 1 and 2).

Löwenmensch: The Lion Man

History of Discovery:

During systematic excavations at the Hohlenstein-Stadel cave in 1937, geologist Völzing and prehistoric historian Wetzel discovered approximately 200 fragments of mammoth ivory that seemed to have been worked upon by human hands. Due to the start of World War II, these fragments were packed away at Ulm Museum in Germany, and forgotten for 32 years.

Archaeologist Hahn began assembling these fragments in 1969. Subsequent restoration activities by multiple experts were carried out in 1982, 1987, 2008, and 2012, and the figurine was completed in late 2013.

Carbon dating puts the age of the figurine at around 40, 000 years ago, which is the end of the last ice age. It is also the era that has been referred to by Steven Mithen as the "big bang of art, science, and religion," which is believed to mark the origins of modern human cognition – that is, these are the earliest people who thought and experienced life the same way we do

Description of Figurine:

The figurine is close to 1 foot in height and depicts a creature with a human body, standing with its legs apart and hands to the side. A long cylindrical torso shades almost imperceptibly into a thick neck and a lion's head. The ears of the lion are cocked, as though it is listening, and intricate detailing around the ear muscles at the back of the figurine confirm that this is not a mask, but rather an actual head of a lion. The stance of the figurine seems to be a powerful one, and the creature seems to display strength.

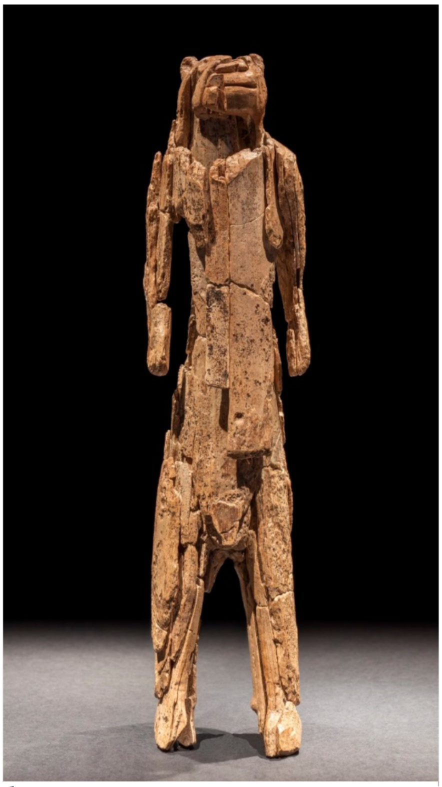

1 Photograph (c) Museum Ulm, photo: Oleg Kuchar, Ulm

Significance of Discovery:

The creation of the figurine would require immense labour and attention to detail, and thus the work of a professional, or at least someone with a lot of proficiency at carving. According to experts, this technically complex figurine would have taken around 400 hours of painstaking work to create. The period of its creation (the end of the last Ice Age), would be one where humans were constantly battling the elements, and

Figure 1: Page 1 of the pamphlet provided to participants.

barely able to sustain themselves. *Why would such a community, living on the edge of subsistence, allow someone to spend this much time on something that does not have any direct functional value?*

This figurine is the **earliest** known example of a physically graspable creation that could not exist in reality. It is therefore an expression of a large imaginative leap, and the earliest example of creativity in the form of combining existing concepts into a completely new idea. *How did the creator of this figurine arrive upon this idea? What inspired them to combine the forms of human and its most dangerous predator, and use the tusk of the largest known mammal to depict it?*

Microscopic analysis has revealed two important details- the inside of the mouth of the lion shows traces of an unidentified organic substance (experts suggest that it might be blood), and the surface of the figure is unnaturally smooth, suggesting that it had been held, passed around, and rubbed by many hands over many, many years. (Although others believe the surface was worn down by flowing water.) In any event, the figurine may have been used for some sort of ceremonial ritual or other significant communal activities. The figurine was also found deep inside the cave, at the very back in a smaller cave-like area, again suggesting both its importance and significance to the humans living in the cave. *What might have been the significance of this figurine to the community? If it was passed from person to person over many years, was it an artefact handed down through generations? What might that mean?*

References

Cook, J. The lion man: An Ice Age masterpiece [Blog Post]. Retrieved from: https://blog.britishmuseum.org/the-lion-man-an-ice-age-masterpiece/

Der Löwenmensch: Die Figur. (n.d.). Retrieved from: http://www.loewenmensch.de/figur_3.html

Kind, C., Ebinger-Rist, M., Wolf, S., Beutelspacher, T., & Wehrberger, K. (2014). The smile of the lion man. Recent excavations in Stadel cave and the restoration of the famous upper Palaeolithic figurine. *Quartar, 61*, 129-145.

Kobrak, P. (Producer). (2017 October 23). The Beginnings of Belief [Audio Podcast]. Retrieved from: https://www.bbc.co.uk/programmes/b099xhmj

Figure 2: Page 2 of the pamphlet provided to participants.

2 ARTISTIC WORKS CREATED FOR THIS STUDY

The following artistic works were created as part of this project.

2.1 MUSIC AND VIDEO BY MICHAEL GARFIELD

A first piece of music and a corresponding video inspired by the Hohlenstein–Stadel Löwenmensch (lion-man) figurine, created this project by Michael Garfield, (Santa Fe, NM, USA), 2020, can be found here: https://www.youtube.com/watch?v=wvOZMT8lqak.
Used with permission of the artist.

2.2 MUSIC BY 'SOUNDS LIKE THINGS ENSEMBLE'

A second piece of music inspired by the Hohlenstein–Stadel Löwenmensch (lion-man) figurine, was created for this project by the Sounds Like Things Ensemble (Kelowna BC, Canada), 2020. Nicholas Denton Protsack - cello, electronics, and found sounds. Andrew Stauffer - percussion instruments and found sounds. Leila Neverland - vocals. The music can be found here:
https://soundslikethings.bandcamp.com/track/tuskarve-roar
Used with permission of the artists.

2.3 POETRY BY LIBBY HATHORN

Löwenmensch: The Lion Man
Libby Hathorn
September 2020

Did you awake then as if from a dream,
turn aside from your punishing labour,
the cold daily struggle of subsistence,
the spark, splinter of ice in your brain,
to fashion this figurine with your precious cutting tool?

Obsessed with making, your feelings heightened
as your understanding
in some sort of act of creation, a bliss hitherto unknown,
as you cut and scraped and thought it into being,
this human figure with its animal head;
a sacramental thing to hold, to venerate,
a piece to command or to frighten?
Anyway, some act of self- love,
a yearning to know newness.

A beat in the brain,
that particular pulse urging creation.
Not the axe, the hammer, the knife.
No, the astounding dream of yourself,
something inherent yet something newborn,
a novel piece created by your hand,
this brave ivory figurine
at the nub of it, intrinsic,
the reaching after your own human core.

Included with permission of the author.

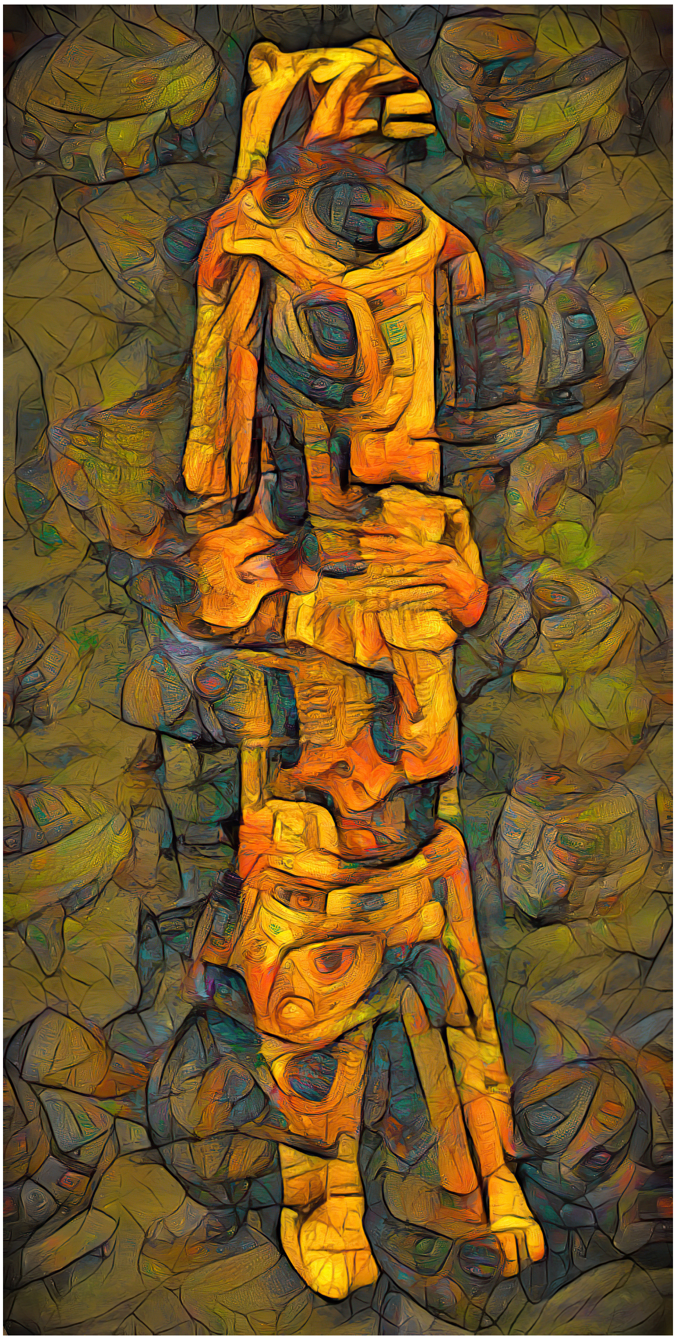

Figure 3: Artistic rendering of the Löwenmensch or 'lion-man' figurine from the Hohlenstein-Stadel cave in Germany by Steve DiPaola (Deep Cove, BC, Canada), 2020. Created using a convolutional deep learning neural network. Used with permission of the artist.

2.4 TWO-DIMENSIONAL ART BY STEVE DIPAOLA

2.5 FICTION BY SUE WOOLFE

This is what happened:

She was born in the beginning of the Great Hunger, in a tribe that lived beside a wide and surging river, so wide that the cleverest ones who ran things argued that the river covered the whole earth except for their narrow strip of land, so there was nowhere to escape to. Some old people were sure that the wind was colder than it used to be, and the rains came less often, and the land was withering and and the animals were starving. But the clever ones who ran things said their land was just going through a few bad seasons and it'd all come good again and no one should attempt to cross the river to what the songs said was a wonderful beyond, because there was no other side. The other side was only made up by silly dreamers. Her mother in her childhood was one of those silly dreamers.

Because food was getting scarce and families were struggling with too many mouths to feed, and the girl had been a strange, uncommunicative child, no one really cared that she'd left the tribe and lived in a cave by herself with only a pet lion cub for company. Only her mother cared, the once-silly dreamers, now beaten down by too many children and too many duties. Often she'd go without, to leave food at the mouth of her daughter's cave. The mother had been married into a family of proud carvers, rather than hunters or clothes makers or other useful skills, and the girl had been trained from babyhood to use a knife. She'd seemed to show great promise as a carver, but because she couldn't be told anything, she'd refused to carve what the tribe needed- cups, axes, bowls for food, water carriers, fire carriers, baby carriers. She'd always disgraced her family by wasting perfectly good ivory by carving it into what was clearly useless. Replicas of trees, plants, heads of family members. What she carved made everyone uneasy. The clever ones argued that the carvings were never as good as what was real, and the waste was terrible. Her parents had to take the messes she'd made and sheepishly try to re-fashion them.

It was said that she and her pet lion existed on little else than plants, roots and small muddy fish from the shallows of the river. They were both skinny, but it was a time when no one's belly was full. She grew into a tall, rangy young woman, constantly in the company of her pet, who was also now fully grown. Living constantly in each other's company, they'd grown more alike. Her eyes had always been yellow, and she'd always had a round, flattened face, and her talk was only to the lion, an odd whinnying sound that meant nothing to anyone else.

Even her father had a grudging respect for his first born because she was so independent, but her eldest brother hated her for that very reason. He was very competitive, the best at everything, the tribe's best carver of useful things only and the best hunter and her most derisive critic, who resented how she'd been indulged. One day he shocked their parents by saying he planned to kill her lion because it was only an animal and would make a fine meal for them all and, as a sop to the parents, that their mother needed the lion's coat. That was a hard argument to resist. He and another brother set out for the kill. The girl and the lion happened to be out gathering roots, and from a mountain top saw the hunters heading towards the cave, and her animal instincts knew to flee with the lion. They were so fleet of foot, it was later claimed that she rode to safety on the back of the lion who could run much faster than any man or woman. The two seemed to disappear without a trace, and the brothers had to return empty-handed, with only a bag of nuts and berries after their fine words. From then on, it was war.

One hot night they eventually surprised the two of them sleeping side by side, outside the mouth of another cave, the girl's arm thrown around her lion. Her brother hurled a spear. The girl awoke and leapt up out of the way, and it was easy for the brothers to slaughter her sleeping pet.

From then on, no one saw the girl for many years. Her mother pined for her lost child. No number of grandchildren made up for her loss.

One day, the girl saw in the distance the aging, broken woman her mother had become, and though every animal instinct told her to stay away, her heart was touched. She knew her mother had once been the maker of songs about the land beyond. During the night, she woke her mother and begged the astonished, tearful woman to bring the family at a particular night when the moon was at its most orange, to a place a long way off. No one was to know why. The mother, herself half thinking it'd been a dream, tried to talk the group into it but old women have very little influence, then or now. All she could say was that she'd dreamed a dream. Only a few believed her dream, young mothers with their children, a few young husbands, her own old husband, his sisters, her sisters. Most of the men ridiculed her, and insisted on

staying behind with her younger brothers and the clever ones. The others set off. It was a long journey for people weakened by poor food and an increasingly cold climate. Some began the journey and then turned back.

The young woman had lived with her lion for so long that she had now the sense and wits of a lion, rather than the silliness of humans. She knew that when the moon was at its largest and most orange, the waters of the river were at their lowest, and she knew where the river's shallows were. She believed in the other side. She could smell it in the wind and sometimes, she could hear it. On the night she'd nominated, she feared her mother would not come. But in the darkness, she smelled the group before she heard or saw them.

The tribe, or the those few who'd come, were terrified when she hailed them, and led them into the dark swirling water. No one could swim. Some refused to walk into it, worrying that something in the water might eat their feet. But she insisted they all hold hands in a long line of people. Still some refused. She warned them that they didn't have much time. They must walk now, or be lost. Soon the waters would deepen. Logic wasn't going to work, but music might, she thought. She began to sing, an animal's strange rendition of her mother's song of the land beyond, and holding her mother's hand and her fathers', and with a child on each shoulder, she strode out singing. The singing gave her mother courage, gave everyone courage. The long line, trembling with courage and fear, walked through the deepening water, fathers and mothers and older siblings all with little ones on their shoulders, walking to what might be their death, but singing.

The water was now up to her mother's neck, but her mother believed in her daughter as she always had, and the song helped her to remember what it was like to be young, and she sang as loud as she ever had, walking and dragging the hands of the others so they'd stay with her. Her husband, inspired by his ancient wife, did the same. At last in the moonlight he cried out above the hubbub of the singing that there was indeed a far bank, and he could see it. No one ever knew if he just shouting that to encourage them. But everyone began to believe in the shore, and strode out. The water was up to their mouths, and they had to stop singing. But then, step by step, the water was shallower and shallower. The song collapsed into shouts. Despite their exhaustion, the people ran to the shore and the shore was muddy, but it was firm. They were on firm land, in the beautiful beyond.

The land was everything the songs had said, and more. From then on, the girl, until her death as an old woman, was celebrated and loved. When it was her time to die, they found in her cave- she'd always lived apart - a carving of a half girl, half lion. Me, the ivory you see. It was as if in me the girl and the lion had finally become one. For generations, I was cradled in many worshipping hands. Because of her, anyone who needed to be an outsider was revered and encouraged. It was remembered that singing had saved them, and music and art by anyone, men and women, were encouraged. I became a cult, the cult of the outsider. But such cults cannot survive for long, for once you begin to create art, you want to do nothing else.

Generations later, the progeny of her brother, competitive, hungry survivors because only the tough could survive in that now terrible climate, eventually heard the singing from the beautiful beyond and found a way to cross the river. Being bred to bitter conflict and hatred, they slaughtered every single soul and, ignoring the fact that they were now living in plenty, ate every soul so that not even a ghost could live. One of the last alive, a musician, knew it was his very last day on the earth. He couldn't bring himself to stop making music but before dawn, he hid me deep in a cave, in the hope that one day, what I'd become would found a new cult of outsiders who believe in art. If anyone has learned to wait, if anything has learned what waiting is, it's me. For forty thousand years, I've waited through kings and pharaohs and empires. A few minutes ago, in my reckoning of time, they at last discovered me in my cave, and I'm waiting for one of my discoverers to hear me. Perhaps you will.

Used with permission of the author.

2.6 WRITING BY SHELLEY BERC

Standing in profile, I am much more handsome. I transform into an insouciant flaneur, slouched against an invisible wall, paws in pockets. Hardly recognizable to you who expect me to be that 14-inch petrified wood fetish. I am really a 40,000 year old ancestor of Fred Astaire or Ginger Rogers, depending which myth you are making me out to be.

Gone is my perfect posture, replaced by a debonair off-kilter tilt; a bodily expression that says ‘I’ve seen it all. All. And I can right myself under any circumstances. I dare you to surprise me’.

If you said to me, Lion God Guy, you were put back together with your original 1000, 40000-year-old fragments of mammoth tusk reconstructed into this 14-inch figurine you in, I would say, ‘That’s ridiculous’.

Now I shall turn myself face forward to prove the point of my great stature. Look, in this position I am regal, tall, and awfully stiff. Hardly the iteration of a wild powerhouse dripping blood and spewing roars that you expect me to be. A powerhouse you want to stuff in your pocket. Really?

Behind these intimidating locked jaws, I am humming a show tune.

There are big black spaces where parts of my brain are missing. As you can see, you can’t miss them. Question—Do these vacancies diminish my cognition or release me to an expanded sense of self? Answer— ‘the latter.’ But I refuse to tell you why.

Being a shape shifter by nature, I need space to take flight. With enough square footage, I have no problem changing from ivory to flesh, from animal to human, man to woman, or vice versa. It is just not an issue for me. I can be as huge and reflexive as a heavy breathing universe or small enough to fit in your hand. My aim is usually the same; to escape the fleshy juice of my gravity and repurpose myself into unending waves of unparticulated undulations rising up and tumbling down until absolutely no one can put me back together again.

It is in the emptiness, the lost parts of this precious statue, where you will find the real me, if, by the way that is what you are after, and most people aren’t.

These days I feel too heavy; I hear the best way to lose weight is to become pure consciousness. I am considering the risk reward of giving up fresh antelope and chips.

What if I was reassembled differently than you chose to do? What if you glued my so-called legs to my shoulder blades for wings or if you simply left me in a pile of the 1000 fragments of me—on a revolving glass stage that every once in a while would shake shake rock n roll, and scatter into different configurations of me. Who wants to look the same every day? I realize you would find that changeability too destabilizing and you might fragment, too. But honestly, that is how we lion-head gods are by nature—we love evaporating and solidifying in new and surprising ways. Afterall, it is bad even dangerous for God types to be bored. So best get used to the winds of change around us. And for God’s sake, stop trying to fit me in your pockets.

Used with permission of the author.

2.7 ANALYSIS OF CREATORS’ RESPONSES

	Direct Quotes	Seen in creative output
	**SLT: Musician Group**	
**LION-HUMAN HYBRID**	We really wanted to focus on the fact that the artist chose a lion and a human.	The background of the piece is the sonically elongated sound of a lion roar<br><br>The piece begins with the 'pure' sound of a lion, and 'human interaction' in the form of instrumentation and rhythm is gradually introduced to interact with this sound
	We will obtain a short audio clip of a lion's roar. We will extract one second of the lion roar sample and stretch the audio file to be a minute or so long	
	We will take turns playing a note or phrase to transform our "raw material" or animalistic roar into something we humans can recognize as "musical", like a rhythmic pattern or a melodic contour, instead of a dense wall of sound.	
	Our human metamorphosis of the lion's roar is inspired by the anthropomorphic nature of this particular statue, as our humanness becomes intertwined with the animalness of the lion.	
	**SW: Writer**	
	I immediately imagined the woman as a misfit whose natural place was with the lion, rather than with other people. I'm a misfit and very much identified with her.	"I *[the figurine]* became a cult, the cult of the outsider"<br>"…the girl *[who is the head of the figurine]* had been a strange, uncommunicative child, no one really cared that she'd left the tribe and lived in a cave by herself with only a pet lion cub for company"
	The head seemed as if it belonged, as if it wasn't odd that a lion's head should be on a woman's body. It took other people to point it out.	
	**WC: Musical Composer**	
	… what else is distinctive is the long thin body with relatively short arms- is it an erect tiger? Because it seems to have club hands and feet, but it doesn't have a tail… it is humanoid	
	The lion as a protector of her offspring (matriarchal goddess worship- female power). *[The figurine]* is a ceremonial object giving protection to her tribe.	

Figure 4: Page 1 of creators' responses to questions organized under throughlines.

	**SLT: Musician Group**	
**SUBTRACTIVE SCULPTING/NEGATIVE SPACE**	This *[the audio of the lion roar]* will be the "raw material" that we work with and will represent the sonic equivalent of an uncarved block, bone, or tusk	The piece begins with a full-bodied sound i.e., a lion roar, and 'degrades' over the course of the piece.<br><br>The sounds become more 'human' (instruments producing recognisable rhythm) towards the end.
	The sound created *[elongation of the lion roar]* will contain many different audible and inaudible pitches. Nick will then use Max MSP software to create a program that will allow us to subtract frequencies from the "raw material," much like reductive sculpting	
	For example, one of us might sing or play an A at 440 Hz. In doing so, the frequencies (fundamental and harmonics) we produce will be removed from the block of sound.	
	This portion of the piece *[sound sculpting]* represents our ability as humans to extract familiarity from an otherwise undifferentiated "raw material."	
	Have each of us of take turns "holding" the song, adding our own layer to it as we "pass it around".	
	As musicians, our canvas is generally silence and we tend to add sound to a blank canvas. In this new form of creation, our canvas is instead sound itself, from which we subtract a variety of sonic material	
	Instead of silence being the 'medium' and sound the 'paint', the opposite had to occur in order for a true analogy to be made to the process of carving. Such a conception of music had, truth be told, never occurred to me before this project.	
	It was like creating negative space instead of positive space.	
	The whole is a balance between the negative and positive.	
	The program Nick wrote … really helped us achieve our vision of 'carving' sound. This could not have been done nearly as effectively without tech/programming.	
	The trees, the ground, the fence, the birds are all descriptors of physical objects that take up positive space. We wondered why we don't give as much importance or value to that which lies between, as in what we call negative space	
	My sister and I realized that the holes *[divets in a garden]* were actually from the left over root bulb of a tree that had been cut down long before I moved in…	

Figure 5: Page 2 of creators' responses to questions organized under throughlines.

	We thought to lay down a massive piece of paper on top of the circumference of the tree root structure, and do a relief drawing. We then realized that the roots had decomposed into what would wind up being seen as negative space on the drawing. We noted that everything the pencil would pick up would be what was initially the space between, as in the negative space between each physically present root. We imagined what the drawing would look like, and concluded that the negative space in the drawing would actually represent the original positive structure of the root bulb.	
	**SLT: Musician Group**	
DETERIORATION / EROSION BY NATURAL FORCES	The next portion of the piece is inspired by the wear and tear of time on a given piece of creative expression, and how the environment interacts with an expression once it is considered "complete" by the original artist.	From the 00:30, we can hear the sounds of water droplets, echoes of sound, as within a cave
	we will use the same program to have the sounds of a cave interact with our creation. We will record the cave for ten or fifteen minutes. The frequency made by an echo, a water droplet, a munching bug will be subtracted from the sound sculpture in the same way that the frequencies played by our instruments were subtracted from the sound wall previously.	
	We anticipate that what we created as our anthropomorphic sound sculpture will deteriorate slowly like the Lion-Man sculpture has over 40,000 years.	The piece reaches a crescendo of instrumentation and slowly deteriorates to silence, mimicking the fate of the figurine as an object actively used by a society and then abandoned in nature for millennia
	We chose to end with this slow deterioration because the art we create is not only the product itself, but how the world and in this case our natural local environment, interacts with it.	
	…. As the song petyrs out the listener is left with an innate concept of what it means for humans to perceive their environment, react to it through creation, interact with creation itself, the natural metamorphosis and deterioration of objects over time…	
	**SW: Writer**	

Figure 6: Page 3 of creators' responses to questions organized under throughlines.

	I found myself suggesting that they *[her students]* imagine running their hands over the figurine as we were told had been done countless times, feeling how smooth the ivory had been worn, imaging [sic] those hands touching her	“For generations, I was cradled in many worshipping hands.”<br>“…hid me *[the figurine]* deep in a cave, in the hope that one day, what I’d become would found a new cult of outsiders who believe in art… For forty thousand years, I’ve waited …”
	**SLT: Musician Group**	
**WAITING (WITH A STORY TO TELL) TO BE FOUND**	… how humans are in a constant state of recreating that which is old into something new.	
	**SW: Writer**	
	Tears jumped to my eyes as I uttered those words *[to listen to the figurine]* because I knew it was speaking right then to me	
	I had a growing conviction that I “knew” the “true” story.<br>I didn’t feel as if I was making anything up, just reporting on what really happened.	
	There’s been something inevitable about this all along.	
	I sat and wrote a draft from the point of view of the ivory, since it felt more and more that the ivory ‘knew’ the story. Like the right character has the voice and “knows” the story when I don’t, and it leads me. To complete a novel which before this ‘knowledge’ is in a million disparate bits.	
	I didn’t feel as if I was making anything up, just reporting on what really happened.	
	What was ‘speaking’ to me was not exactly the figurine, but the ivory. The DNA of the mammoth inside the ivory… I just wrote down the ivory’s thoughts	“When it was her time to die, they found in her cave- she’d always lived apart – a carving of a half girl, half lion. Me”

Figure 7: Page 4 of creators’ responses to questions organized under throughlines.

	I know rationally that Ivory couldn't have spoken to me, but I have an inner conviction otherwise. I've been writing from a country of truth.	
	[experienced emotion of] Relief. I feel that I've done "right" by the ivory	
	**WC: Musical Composer**	
	Why did she end up in this cave? What catastrophic event brought her the state she is in? Who broke her? How did they break her?	
	Finding *[the figurine]* shattered in a cave not considered habitable to humans (i.e., dark and north-facing) with no other objects suggests that something happened in which the offspring/tribe were devastated and lost their faith in the lioness & destroyed her in despair & took her far away from their homes so she was cast into oblivion where she stayed for all these years.	

Figure 8: Page 5 of creators' responses to questions organized under throughlines.
